# The relationship of anxiety, depression and behavioral problems with recurrent headache in late adolescence – a Young-HUNT follow-up study

**DOI:** 10.1186/1129-2377-16-10

**Published:** 2015-01-16

**Authors:** Brit A Blaauw, Grete Dyb, Knut Hagen, Turid L Holmen, Mattias Linde, Tore Wentzel-Larsen, John-Anker Zwart

**Affiliations:** Department of Neurology, Vestfold Hospital, Tønsberg, Norway; Faculty of medicine, Institute of Clinical Medicine, University of Oslo, Oslo, Norway; Norwegian Centre for Violence and Traumatic Stress Studies, Oslo, Norway; Department of Neuroscience, Faculty of Medicine, Norwegian University of Science and Technology, Trondheim, Norway; Norwegian National Headache Centre, Department of Neurology and Neurophysiology, St. Olavs Hospital, Trondheim, Norway; HUNT research Centre, Department of Public Health and General Practice, Faculty of Medicine, Norwegian University of Science and Technology, Trondheim, Norway; Center for Child and Adolescent Mental Health, Eastern and Southern Norway, Oslo, Norway; Department of Neurology and FORMI, Oslo University Hospital, Ullevål, Oslo, Norway; Sykehuset I Vestfold HF, Tønsberg, 3103 Norway

**Keywords:** Recurrent headache, Migraine, Tension-type headache, Anxiety, Depression, Behavioral problems, Conduct difficulties, Attention difficulties, Adolescents

## Abstract

**Background:**

The comorbidity of headache and psychiatric symptoms is a well-recognized clinical phenomenon, but there are only limited data regarding the temporal relationship between headache and symptoms of anxiety and depression as well as behavioral problems in adolescents. This study investigates the relationship of anxiety and depressive symptoms and behavioral problems at baseline with recurrent headache at follow-up four years later.

**Methods:**

Within the Nord-Trøndelag Health Study (HUNT), including repeated population-based studies conducted in Norway, 2399 adolescents in junior high schools aged 12–16 years (77% of the invited) participated in Young-HUNT1 (1995–1997) and again at follow-up four years later, in Young-HUNT2 (2000–2001). The same comprehensive questionnaire that included assessment of symptoms of anxiety and depression and behavioral problems, i.e. conduct and attention difficulties was completed in both studies. In addition 1665 of the participants were interviewed about their headache complaints in Young-HUNT2.

**Results:**

In adjusted multivariate analyses we found that higher scores of anxiety and depressive symptoms at baseline were associated with recurrent headache at follow-up four years later (OR: 1.6, 95% CI: 1.2-2.1, *p* = 0.001), evident for migraine (OR: 1.8, 95% CI: 1.2-2.7, *p* = 0.008) and non-classifiable headache (OR: 1.7, 95% CI: 1.0-2.8, *p* = 0.034), but not statistically significant for tension-type headache (OR: 1.4, 95% CI: 1.0-1.9, *p* = 0.053). Higher scores of anxiety and depressive symptoms at baseline were significantly associated with more frequent headache at follow-up (monthly vs. no recurrent headache OR: 1.8, 95% CI: 1.3-2.5, *p* = 0.001, weekly or daily vs. no recurrent headache OR: 1.9, 95% CI: 1.2-2.9, *p* = 0.005). Among adolescents without recurrent headache at baseline, higher scores for symptoms of anxiety and depression were associated with new onset migraine four years later (OR: 2.6, 95% CI: 1.1-4.8, *p* = 0.036). Higher scores of attention problems at baseline were associated with non-classifiable headache at follow-up (OR: 2.0, 95% CI: 1.3-3.4, *p* = 0.017).

**Conclusions:**

Results from the present study showed that symptoms of anxiety and depression in early adolescence were associated with recurrent headache four years later. Recognizing anxiety and depressive symptoms should be considered part of the clinical assessment in young headache patients, as early identification of these associated factors may lead to improved headache management.

## Background

It is documented that recurrent headache is one of the most common medical complaints in adolescence, with an estimated prevalence of approximately 10% for migraine and 15-20% for tension-type headache (TTH) in population-based studies [1–6]. Population-based cross-sectional studies in children and adolescents have shown that recurrent headache is associated with anxiety and depression, and also with attention and conduct difficulties [7, 8]. Results from several longitudinal studies among adults suggest that the association between depression and headache may be bi-directional [9–11]. There is, however, uncertainty in the literature regarding the direction of the relationship between headache and psychiatric symptoms among adolescents [12, 13]. The clinic-based studies are not necessarily representative of all adolescents with recurrent headache due to selection bias and there are challenges when comparing population-based longitudinal studies because of inconsistencies in their methodological approaches [13, 5]. There is evidence that suggests that psychiatric symptoms are more common among those with chronic daily headache [14, 15], but whether this comorbidity is more specifically related to migraine than to TTH is uncertain [7, 16].

Recurrent headache represents a significant health problem among adolescents [17, 18] and can affect all aspects of adolescents functioning including negative affective states and increased psychosocial problems. It is even reported higher suicidal ideation among adolescents with migraine compared with non-migraine subjects [19]. It is known that comorbid psychological symptoms generally portend a poorer outcome for headache management [13], but the nature of this relationship among adolescents remains unclear. In order to develop prevention strategies it is important to improve the understanding of headache evolvement through adulthood and try to identify risk factors. To study the epidemiology and comorbidity of headache it is necessary to collect subjects from an unselected population. Conducting repeated studies of headache symptoms in the same population is a reliable method of assessing changes over time and may support causal interference. The objective of the present study was to identify associations between symptoms of anxiety and depression, conduct difficulties and attention difficulties in early adolescence and recurrent headache four years later.

## Methods

### Young-HUNT1 and 2

During a two-year period from August 1995 to June 1997, all students in lower secondary school and upper secondary school (aged 12 to 20 years) in Nord-Trøndelag County in Norway were invited to participate in the youth part of the Nord-Trøndelag Health Study, Young-HUNT1. A detailed description of the study has been published previously [1, 20].

In the school years 1999–2000 and 2000–2001 a follow-up study of Young-HUNT1 was performed. All students in 2nd and 3rd grade in upper secondary school (aged 16 to 20 years) in Nord-Trøndelag County and all apprentices of the same age who participated in Young-HUNT1 were invited. It was a replicate study, with the same questionnaire, clinic examination and headache interview as in Young-HUNT1. In total, 2399 (81%) of the invited adolescents completed the questionnaire in Young-HUNT2 and 1665 adolescents underwent a headache interview. The clinic examination and headache interview were done at school in school hours. The apprentices did not attend to school, thus only students were able to participate in this part of the study and explains the lower number of interviewed subjects. Out of the interviewed participants, 1586 had answered all questions regarding symptoms of anxiety and depression and behavioral problems in the questionnaire in Young-HUNT1 and were included in the analyses. Among these 1586 adolescents, headache status based on interview in Young-HUNT1 was known for 1194 participants.

The headache interview was done in connection with a clinical examination performed at the schools during school hours. Trained nurses performed the interviews, and the students were asked if they in the past 12 months had experienced recurring headaches that were not related to cold, fever or any other disease. Two typical headache symptom history descriptions, one for migraine and one for tension-type headache were then read to those students who reported having had recurrent headache, and they were asked to classify their headache according to the descriptions. The students were also given a third alternative (“non-classifiable headache”) in case neither of the two descriptions resembled their own symptoms. Headache frequency during the past year was recorded according to the following categories: less than 1 day per month (less than monthly), 1–3 days per month (monthly), 1–5 days per week (weekly), or more than 5 days per week (daily) [1, 21].

The “recognition-based” headache diagnoses obtained by the nurses contained typical features for both migraine and TTH in accordance with the International Headache Society (IHS) criteria, and have previously been validated against extensive semi structured interviews by neurologists [22].

The comprehensive self-administered questionnaire included several health-related questions and was completed during one school hour, in an exam setting. Symptom Check List (SCL-5) was used to measure symptoms of anxiety and depression. This short form checklist contains five questions and is based on statistical analyses of 23 of the items in the more extensive SCL-25 and is proven reliable in previous studies [23, 24]. The items concerning anxiety symptoms were: Been constantly scared and uneasy, felt tense and restless, worried too much about different matters. Depressive symptoms: felt hopeless when thinking of the future, felt down or sad. All questions had four alternative responses ranging from one: “not at all” to four: “extremely”. The SCL-5 is not an appropriate diagnostic instrument for differentiating anxiety and depression, thus the five item SCL-5 score was defined as an anxiety/depressive variable. A mean score of the SCL-5 questions was computed, giving a scale ranging from one to four. In the present study the SCL-5 reached a Cronbach’s alpha of 0.79.

Variables concerning behavioral problems (attention- and conduct difficulties) were derived from the school adjustment part of the questionnaire with totally 14 items, described in previous studies [25, 26]. The adolescents were asked: “Do any of these situations happen to you at school, or have they happened before?” Attention difficulties were estimated by the question how often “do you have problems concentrating in class”, conduct difficulties included how often the participants “quarrel with the teacher”, “get into fights” and “get scolded by the teacher”. The students were given four alternative responses ranging from one: “never” to four: “very often”. A mean score ranging from one to four was computed. Cronbach’s alpha for conduct difficulties was 0.62.

### Ethics

Participation in the study was voluntary and based on written statements of consent from all participants. In addition, written consent from the parents was collected for students below 16 years of age. The Regional Committee for Medical and Health Research Ethics and the Norwegian Data Inspectorate Board approved the study.

### Statistical analyses

The associations of anxiety and depressive symptoms as well as behavioral problems at baseline with headache status at follow-up were estimated using logistic regression. Recurrent headache or not in Young-HUNT2 was used as dependent variable in the main analysis. In supplementary analyses migraine, TTH and non-classifiable headache compared to no recurrent headache in Young-HUNT2 were used as dependent variables. The independent variables were included by hierarchic regression. Symptoms of anxiety and depression in Young-HUNT1 were added in the first step, attention difficulties in Young-HUNT1 in the second step and conduct difficulties in Young-HUNT1 in the third step. The same kinds of analyses were done in the sub-sample of Young-HUNT1 without recurrent headache, with new-onset headache versus no recurrent headache in Young-HUNT2 as dependent variable. All analyses were adjusted for sex and age and also for living condition and single parenthood in Young-HUNT1 (“no” if living together with both parents, otherwise “yes”). Data analyses were performed with the SPSS, version 20.0 (SPSS, Chicago, IL).

## Results

Recurrent headache in Young-HUNT2 was reported by 557 (35.1%) out of 1586 participants who had answered all questions about anxiety and depression and behavioral problems in Young-HUNT1, 391 girls (45.7%) and 166 boys (22.7%). Mean scores for symptoms of anxiety and depression and behavioral problems at baseline related to headache status at follow-up are shown in Table 1. The adjusted logistic regression analyses showed that higher scores of anxiety and depressive symptoms at baseline were significantly associated with recurrent headache in total at follow-up (OR: 1.6, 95% CI: 1.2-2.1, *p* = 0.001). As shown in Table 2, there was a significant association between higher scores of anxiety and depressive symptoms in Young-HUNT1 with both migraine and non-classifiable headache in Young-HUNT2, but not with TTH. There was not any significant association between behavioral problems at baseline and recurrent headache in total or with different headache types at follow-up.

**Table 1:** **Levels of anxiety and depressive symptoms, attention difficulties and conduct difficulties in girls and boys in Young-HUNT1 in relation to headache status in Young-HUNT2**

	Boys						Girls					
	Total (n = 730)		Recurrent headache (n = 166)		Headache-free (n = 564)		Total (n = 856)		Recurrent headache (n = 391)		Headache-free (n = 465)	
	Mean	SD	Mean	SD	Mean	SD	Mean	SD	Mean	SD	Mean	SD
Age	14.20	0.77	14.14	0.79	14.21	0.76	14.25	0.78	14.25	0.80	14.25	0.77
Anxiety and depressive symptoms (1–4)	1.27	0.37	1.33	0.41	1.25	0.35	1.42	0.43	1.46	0.44	1.38	0.43
Attention difficulties (1–4)	1.94	0.63	2.01	0.70	1.91	0.61	2.04	0.61	2.06	0.58	2.03	0.64
Conduct difficulties (1–4)	1.44	0.43	1.44	0.42	1.44	0.43	1.29	0.33	1.30	0.33	1.29	0.33

*Abbreviations: n* number, *SD* standard deviation.

**Table 2:** **Associations of anxiety and depressive symptoms and behavioral problems in Young-HUNT1 with recurrent headache and headache diagnoses in Young-HUNT2**

	Recurrent headache (yes = 557, no = 1029)		Migraine (yes = 136, no = 1029)		Tension-type headache (yes = 316, no = 1029)		Non-classifiable headache (yes = 105, no = 1029)	
	OR (95% CI)	***p***-value	OR (95% CI)	***p***-value	OR (95% CI)	***p***-value	OR (95% CI)	***p***-value
***Step 1***								
Symptoms of anxiety and depression (1–4)	1.60 (1.23-2.07)	<0.001	1.84 (1.24-2.71)	0.003	1.40 (1.03-1.90)	0.032	1.67 (1.07-2.62)	0.024
***Step 2***								
Symptoms of anxiety and depression (1–4)	1.56 (1.19-2.05)	0.001	1.79 (1.17-2.74)	0.007	1.37 (0.99-1.91)	0.057	1.68 (1.03-2.74)	0.037
Attention difficulties (1–4)	1.05 (0.87-1.26)	0.627	1.05 (0.77-1.43)	0.752	1.04 (0.83-1.30)	0.760	0.99 (0.71-1.39)	0.962
***Step 3***								
Symptoms of anxiety and depression (1–4)	1.57 (1.19-2.07)	0.001	1.79 (1.17-2.74)	0.008	1.39 (1.00-1.93)	0.053	1.70 (1.04-2.77)	0.034
Attention difficulties (1–4)	1.06 (0.88-1.29)	0.523	1.05 (0.76-1.44)	0.769	1.06 (0.83-1.34)	0.655	1.02 (0.72-1.45)	0.919
Conduct difficulties (1–4)	0.91 (0.67-1.24)	0.546	1.01 (0.60-1.69)	0.972	0.90 (0.61-1.32)	0.590	0.85 (0.46-1.55)	0.584

*Abbreviations: OR* odds ratio, *CI* confidence interval.

All analyses are adjusted for age, sex and single parenthood.

Higher scores of anxiety and depressive symptoms at baseline were significantly associated with more frequent headache at follow-up, monthly vs. no recurrent headache OR: 1.8, 95% CI: 1.3-2.5, *p* = 0.001, and weekly or daily vs. no recurrent headache OR: 1.9, 95% CI: 1.2-2.9, *p* = 0.005. Higher scores of conduct difficulties in Young-HUNT1 were significantly associated with weekly or daily headache in Young-HUNT2 OR: 1,8, 95% CI: 1.0-3.0, *p* = 0.048. There was no significant association between higher scores of anxiety and depressive symptoms and behavioral problems with having less than monthly headache.

Among the 1194 participants with known headache status (interviewed) in Young-HUNT1, 882 adolescents reported no recurrent headache at baseline. In this subsample, mean scores for symptoms of anxiety and depression were 1.30 (SD 0.38), attention difficulties 1.95 (SD 0.60) and conduct difficulties 1.37 (SD 0.40). At follow-up 215 (24.4%) had developed recurrent headache (4.7% migraine, 15.0% TTH and 4.8% non classifiable headache). Analyzing this population with logistic regression (adjusted for age, sex and socio demographic factors) we found that higher scores of anxiety and depressive symptoms were significantly associated with new onset migraine four years later (OR: 2.3, 95% CI: 1.1-4.9, *p* = 0.032). Higher scores of attention difficulties at baseline were significantly associated with new onset non-classifiable headache at follow-up (OR: 2.0, 95% CI: 1.1-3.4, *p* = 0.017).

## Discussion

This is the first large-scale population-based follow-up study in Norway assessing the association of recurrent headache with anxiety and depressive symptoms as well as behavioral problems among adolescents. In the present study symptoms of anxiety and depression at baseline were associated with recurrent headache at follow-up. We did not find the same association regarding attention difficulties and conduct difficulties. Among adolescents without recurrent headache at baseline, symptoms of anxiety and depression were associated with new onset migraine four years later and attention difficulties at baseline were associated with new onset non-classifiable headache.

Comorbidity of migraine and psychiatric symptoms has been extensively studied, but the mechanisms underlying this phenomenon are far from clear. There are several possible mechanisms of comorbidity: The association of two disorders may be a result of chance, one disorder may cause the other or shared environmental and/or genetic risk factors may underlie both disorders. Although population-based cross-sectional studies among adolescents have consistently reported an association between migraine and psychiatric symptoms [8, 12], results from longitudinal studies are often inconsistent and the temporal relationship is less documented. Studies of psychopathology and headache have demonstrated shared neuropathic mechanisms between migraine and affective disorders, involving limbic activation [27, 28]. Disturbances in serotonergic systems are found in completed and attempted suicides, and the serotonergic systems are involved in the pathophysiology of both migraine and affective disorders [29]. Positron emission tomography and functional magnetic resonance techniques have shown that pain and mental suffering are related to the same brain regions and these data indicate that pain and mental suffering share a common basis [29]. In addition, white matter hyperintensities on magnetic resonance images have been associated with both mood disorders and chronic migraine [30]. Even though not completely understood, these finding suggest that shared environmental and/or genetic risk factors may underlie both migraine and psychiatric symptoms, and may explain the correlation between chronic headache, poor quality of life and suicidal risk.

As in earlier population-based studies, cross-sectional data from Young-HUNT1 showed that recurrent headache was associated with symptoms of anxiety and depression among adolescents aged 12–14 years and 15–17 years [21]. A meta-analysis of studies investigating the association of migraine and depression among adults, showed that depression was almost two times more frequent in subjects with migraine than in people unaffected by headache and supported the view that migraine and psychiatric comorbidity are bi-directionally linked [31]. Several earlier longitudinal studies have shown preceding anxiety or depression as a risk factor for new onset migraine [32, 33, 9, 34, 10], while others have not [35, 36]. A recent population-based longitudinal study of major depression and migraine showed that respondents with major depressive episodes were 40% more likely to develop migraine. The association, however, was no longer significant after adjustment for stress and childhood trauma (parental divorce, a lengthy hospital stay, prolonged parental unemployment, frequent parental alcohol, or drug use) [37]. There are only a limited number of longitudinal studies evaluating symptoms of anxiety and depression as a risk factor for non-migrainous headache, and they have not been able to show a significant association [33, 34, 10].

Regarding the association between behavioral problems and recurrent headache, cross-sectional studies have shown diverging results and few longitudinal studies are performed. According to a systematic review of clinical studies on psychopathology in children and adolescents with migraine, there is no evidence that attention problems and externalizing symptoms are more frequent in this group compared with headache free controls [38]. In contrast, a meta-analysis based on both clinical and population studies reports higher scores in externalizing symptoms (mainly behavioral problems) among migraine patients, but not for TTH when compared to healthy controls [39]. In the present study we could not identify attention difficulties or conduct difficulties as risk factors for recurrent headache, but in adolescents without recurrent headache at baseline, attention difficulties were related to an increased risk of new onset non-classifiable headache. The differing diagnostic criteria used, is likely an important reason for the variability in estimates of psychopathology and headache in the pediatric and adolescent population.

For headache frequency, symptoms of anxiety and depression and conduct difficulties at baseline were associated with more frequent headache at follow-up. This suggests that that the level of psychiatric symptoms might be more related to the frequency and severity of headache than to headache subtype. It is previously reported higher prevalence of comorbid psychiatric disorders in chronic daily headache than in other headache subtypes both in children, adolescents and adults [15, 14]. Whether this association is specifically linked to recurrent headache, or to headache as a chronic pain is unclear.

The strengths of the study include the large and unselected population, the relatively high participation rate, and the use of validated headache diagnoses. There was no systematic selection of the interviewed participants and headache diagnoses were assessed at baseline and at follow-up by the same clinically validated approach. Answering identical questionnaires repeatedly during the HUNT surveys may have affected the participants’ responses to the questions. Although we used a validated “recognition-based” headache diagnostic method in our study, misclassification may have had an impact on the supplementary analyses. The questionnaires that were used to measure symptoms of anxiety and depression and behavioral problems have well known advantages as well as limitations [23]. The fact that the psychological symptoms are based on self-report measures may be associated with less reliable answers and possible underreporting. A questionnaire method does not produce any clinical diagnosis [40], which is one of the important limitations of the present study.

## Conclusion

Our results confirm that anxiety and depressive symptoms in adolescents are associated with recurrent headache, in particular migraine. Clinicians should be aware of this relationship as it portends a poorer outcome for headache management and the disability and burden of these disorders may be compounded when present together. We have not controlled for stress and childhood trauma in our study, but it is likely that these are common risk factors for both recurrent headache and psychological symptoms and should be taken in account in later studies. Early identification and management of risk factors is important and may lead to better headache treatment and more effective strategies to prevent chronification of headache. This might have considerable benefits for the patient and the society, a question in need of further research.
